# Burnout and safety outcomes - a cross-sectional nationwide survey of EMS-workers in Germany

**DOI:** 10.1186/s12873-018-0177-2

**Published:** 2018-08-20

**Authors:** Natalie Baier, Karsten Roth, Susanne Felgner, Cornelia Henschke

**Affiliations:** 1 Institute of Health Care Management, Berlin Centre of Health Economics Research (BerlinHECOR), Technische Universität Berlin, Straße des 17. Juni 135 (H80), 10623 Berlin, Germany; 2 ASB Köln e.V, Sülzburgstraße 146, 050937 Köln, Germany

**Keywords:** EMS, Prehospital, Burnout, Safety outcomes

## Abstract

**Background:**

The association between burnout and patient safety has been analyzed in many studies for nurses, physicians, and residents. However, studies concerning prehospital emergency medical services (EMS) workers are limited, although they are particularly under risk for emotional stress. This study aims to descriptively analyze the overall degree of burnout among EMS-workers, and potential adverse events that might harm patients as well as the relationship between burnout and perceived safety outcomes for EMS-workers in Germany.

**Methods:**

EMS-workers were recruited via German EMS-journals, social media and a professional association to participate in an online survey. The questionnaire includes the ´Maslach Burnout Inventory´ (MBI), the ‘Emergency Medical Services Safety Inventory’ (EMS-SI), and items about job satisfaction and the individual person. Data was descriptively analyzed by calculating frequencies, means, percentages and Pearson correlation coefficients. The association between burnout and patient safety was analyzed using linear and logistic regression models.

**Results:**

A total of *n* = 1101 questionnaires were considered for data analysis. The vast majority of participants were male, younger than 40 years old, and full-time employees with an EMS-experience of 12 years on average. Between 19.9 and 40% of the participants showed a high degree of burnout in one of the burnout dimensions. Safety compromising behavior was the outcome measure with the highest percentage of participants reporting a negative outcome measure. The dimensions emotional exhaustion and depersonalization were positively associated with the safety outcomes injury and safety compromising behavior. Additionally, experiences, job satisfaction and the intention to leave the current job were significantly associated with the outcome measures.

**Conclusion:**

This is the first study that examines the association between the degree of burnout and patient safety for EMS-workers. The results suggest that an expansion of psychological support for EMS-workers should be considered. Further research should concentrate on the complex relations between working conditions, burnout and patient safety.

**Electronic supplementary material:**

The online version of this article (10.1186/s12873-018-0177-2) contains supplementary material, which is available to authorized users.

## Background

Psychological burden of health care professionals is an intensively discussed topic, especially in the context of potential negative consequences for patients. It is in the nature of these professions that they have a high risk for psychological diseases as they are exposed to situations with high emotional stress levels. Persistent emotional stress might lead to the burnout syndrome [1], first mentioned by Herbert Freudenberger for helping professions which sacrifice themselves for others resulting in a burnout [2, 3]. Maslach and Jackson [1] conceptualized the burnout syndrome by three dimensions: emotional exhaustion, depersonalization and personal accomplishment [1] which are the basis of the Maslach Burnout Inventory, the most commonly used self-assessment instrument to evaluate the degree of burnout [4]. Based on this conceptualization, there are different results for the prevalence of burnout with regard to the considered medical profession. Prevalence for emotional exhaustion ranges from 27% (nurses [5]) and 47% (physicians from all specialty disciplines [6]) to 57% (residents [7]) of the study participants and for depersonalization from 10% (nurses [5]) and 35% (physicians from all specialty disciplines [6, 8]) to 50% (residents [7]) of the study participants.

Following Leiter [9], employees that experience burnout (emotional exhaustion) a more likely to move away from people associated with their jobs including the patients. Based on an imbalance between investment of work resources and return of investment, employees or health care providers will be more reserved regarding future investments and may develop negative attitudes towards their patients [10]. This conservation of resources can have a negative impact on the patient safety. The association between burnout and patient safety has been examined in many studies focusing on physicians and nurses [11–13]. A systematic review by Hall et al. [11] found a significant association between burnout and patient safety for 24 studies (*n* = 30). However, EMS-workers (providing care out-of-hospital) are only examined in a few studies although they are particularly under risk for emotional stress as they often provide care in public environments with a high potential of traumatizing effects. National and international studies of EMS-workers focused on the correlation between burnout and organizational working conditions [14], coping strategies [15], job related stress factors [16, 17] and/or the exposure to critical incidents as a child victim or particularly severe injuries [18]. Only one study by Hammer et al. [19] focused on patient safety measured by judgement errors in patient care and its association with wellbeing measures as job satisfaction and somatic distress for paramedics in the US.

However, the relation between burnout and patient safety has not been analyzed for EMS-workers so far. Although, research on this association is imperative in light of workforce shortages e.g. due to high rates of sick leave and an increasing number of cases that cause growing concerns regarding the prehospital emergency care in Germany and other developed countries [20–22]. This study aims to fill this research gap by (1) evaluating the frequency of self-perceived negative safety outcomes and the prevalence of burnout among EMS-workers and (2) analyzing the association between the degree of burnout and patient safety for EMS-workers in Germany.

## Method

### Recruitment of participants and data collection

EMS-workers were recruited via call for joining the survey at social media channels including specialized groups at Facebook and Twitter, German EMS-journals (´Rettungsdienst´ and ´retten!´), and the professional association for non-medical EMS-workers (´Deutscher Berufsverband Rettungsdienst e.V.´). The main reason for this recruitment was to avoid employer-biased responses, which might have occurred when recruiting EMS-workers via their employers. Three reminders were used in social media after one, two, and four months. EMS-workers in this study are defined as trained professionals in prehospital emergency medical care according to their level of qualification excluding emergency physicians. Their tasks cover medical care on-site and in ambulances as well as the transport of patients to hospitals.

With the aim of testing the comprehensibility of the instructions and the appropriateness concerning the order of questions in the questionnaire, a pilot survey including 10 EMS-workers was conducted. As a result, some minor changes regarding the order of questions were implemented before starting the large-scale survey. Data collection was performed from June to December 2015 using the online survey tool SoSciSurvey, which records data according to the German privacy policy. The study makes use of a cross-sectional design by conducting a nationwide survey to EMS-workers.

### Instrument

The final survey consists of the Maslach Burnout Inventory - Human Services Survey (MBI-HSS), the EMS-Safety Inventory (EMS-SI), accompanied by the EMS-Safety Attitudes Questionnaire (EMS-SAQ) as well as items of job satisfaction adapted from the nurse survey within the project Nurse forecasting in Europe (RN4Cast) [23], and items to collect individual data of the respondents, e.g. socioeconomic characteristics. Most items of the RN4Cast study (inclusion of questions regarding satisfaction) and all items of the EMS-SAQ are not considered in this study as they are not focused in this study or were collected for a planned comparative study with nurses. The MBI – HSS uses a total of 22 items grouped in three dimensions to examine the degree of burnout [24]. Each statement is rated according to the frequency of occurrence adapting a seven-point Likert scale [1]. The subscale emotional exhaustion (EE) assesses feelings according to an emotional overextension, the exhaustion in someone’s work or the contact with other people. The subscale depersonalization (DP) measures the callousness and impersonal response towards people receiving service, care, treatment, or instruction. The subscale personal accomplishment (PA) assesses feelings of being competent and successful in working with people [1, 24]. Specific sum scores of the items per dimension indicate the degree of burnout, ranging from ‘high’ to ‘low’. Based on the manual of Maslach et al. [24] respondents are assigned to a high degree of burnout with a sum score ≥ 27 in the subscale EE, ≥ 13 in DP and ≤ 31 in PA [24]. In line with current studies [6, 25, 26], high scores for emotional exhaustion and depersonalization were additionally considered.

The EMS-SI has been used for identifying and rating safety outcomes in EMS. This self-reporting instrument is seen as an alternative to incomplete and inaccurate data of electronic patient care reports [27, 28]. It includes 44 items within three safety outcome measures: ‘provider injury’, ‘error or adverse events’, and ‘safety-compromising behaviors’ as these issues bear the risk of harming providers and patients [29]. Two nominal, seven-option, categorical scales were used (A – 35 items, B – 19 items) for extracting answers which have to be referred to the previous three months of working [27].

The demographic section includes years of working in EMS, current EMS-area, highest apprenticeship, employment relationship and federal state of participants’ workplace. In addition, the respondents were asked about their age when reaching the highest level of EMS-education, satisfaction with occupational choice (6-point Likert scale), and the operating ranges.

As the MBI was translated into German language by the study group of the project RN4CAST, no further adaption was necessary. The EMS-SAQ and EMS-SI used for the first time in the German context were translated using the back translation method [30]. The original and translated EMS-SAQ and EMS-SI as well as details regarding the items of each instrument, used scales and corresponding dimensions/categories are available in the Additional file 1.

### Analysis of the data

#### Data screening process

The target population were EMS-workers actively working in their position at the time of the survey period. Before starting the survey, participants had to approve a data privacy statement including (1) details about the content and duration of the survey, (2) indications that participation may evoke negative experiences, and (3) information on the possibility to interrupt the survey at any time. The last point includes the possibility to withdraw participants’ data. In this context, participants received an e-mail address assuring that their data can be deleted anytime. Therefore, they had to enter an eight-digit code. Missing approvals of the data privacy statement and the eight-digit code led to the exclusion of participants. For those participants who opened the survey link, the following criteria resulted in an exclusion of the questionnaires: (1) multiple records, and (2) questionnaires, where less than 50% of the items are completed. Implausible answers as typing errors for single questions (*n* = 10) were handled like missing data and substituted applying unconditional mean imputation [24].

#### Statistical analysis

The MBI, EMS-SI and the participants’ characteristics were descriptively analyzed on the respondent-level using the manuals of the different instruments. Percentages and frequencies were calculated for the MBI and the EMS-SI. Frequencies, percentages, means, their corresponding standard deviations (SD) and min/max were examined for the participant characteristics. The Fisher exact test was used to compare the dimensions of the MBI between EMS workers who reported negative safety outcomes and EMS workers who did not report negative safety outcomes. Additionally, the association between the EMS-SI und the MBI was analyzed using the Pearson correlation coefficient. Linear and logistic regression models were used to evaluate the association between the dimension of burnout and the three safety outcomes. The dimension personal accomplishment was not included in the linear regression models as several studies have questioned the validity of this dimension [31, 32]. For the regression analysis all results with a *p*-value < 0.1 were considered significant, for the Pearson correlation coefficient and for the Fisher exact test all result with a p-value < 0.01. Analyses were performed using the statistical software package Stata 12.

## Results

### Participants’ characteristics

Based on the data screening process described in the method section, 1101 questionnaires could be considered for the data analysis.

The majority of all participants were male (86.2%) and younger than 40 years old (73.2%). On average, the participants had an experience in EMS-work of 12 years. Experiences in their current EMS-area comprised 9 years. The questions regarding the level of the participants’ EMS-qualification include the German terms of occupational profiles (See Additional file 2 for detailed description on EMS-qualification). The majority of the respondents (85.3%) had the qualification level of a paramedic and worked in a full-time position (89.6%). Half of the respondents (53.7%) were satisfied with their current job, 24% of the respondents with their wages and 33% with their professional status. No intention to leave their current job due to dissatisfaction was reported by 45% of the respondents. Nearly half of the respondents (46.2%) would recommend their current EMS area as a good place to work. Participants’ demographic and occupational characteristics are outlined in Table 1.

**Table 1:** Characteristics of the study participants

Participants; *n* = 1101 (%) or (SD/min – max)	
Gender	
Male	949 (86.2)
Female	152 (13.8)
Age in years	
≤ 29	439 (39.9)
30–39	367 (33.3)
40–49	205 (18.6)
≥ 50	90 (8.2)
Experience in EMS in years	
Mean	12.34 (9.11/0.5–42)
Experience in current EMS-area in years	
Mean	9.01 (8.22/0–42)
Level of EMS-qualification*	
Paramedic ‘Notfallsanitäter’	121 (11.0)
Paramedic ‘Rettungsassistent’	818 (74.3)
EMT-I ‘Rettungssanitäter’	115 (10.4)
EMT-B ‘Rettungshelfer’	4 (0.4)
Other	43 (3.9)
Employment relationship	
Full-time	986 (89.6)
Part-time	94 (8.5)
Voluntary	21 (1.9)
Satisfaction	
Satisfaction in the current job	591 (53.7)
Satisfaction with wages	266 (24.2)
Satisfaction with the professional status	371 (33.7)
Intention to leave/Recommendation	
No intention to leave the current job within the next year as a result of job dissatisfaction	500 (45.4)
Recommendation of the current EMS-area as a good place to work	509 (46.2)

Caption: *B* basic, *EMS* emergency medical services, *EMT* emergency medical technician, *I* intermediate

* A more detailed description on EMS-qualification can be found in the Additional file 1: Appendix B

### Descriptive results regarding the degree of burnout and safety outcomes

**Fig. 1:**
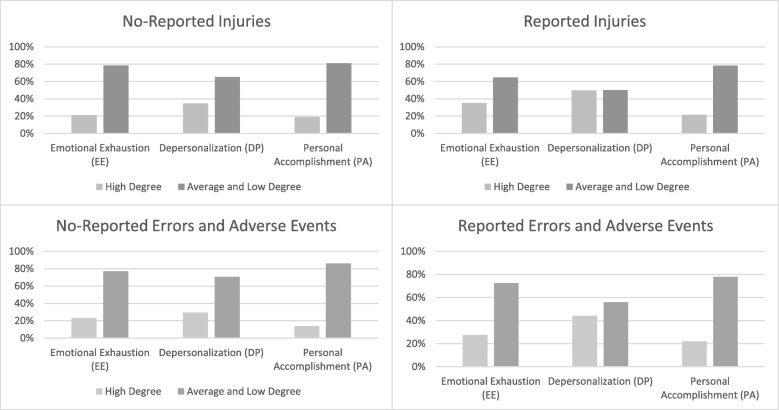
Degree of burnout for reported and not reported negative safety outcomes

Based on the cut-off point presented in the method section the answers of the participants regarding the three dimensions of the MBI were categorized in high degree and low degree of burnout. High degrees could be shown for 25.3% (*n* = 290) of the participants in EE, for 40.2% (*n* = 443) of the participants in DP and 19.9% (*n* = 219) in PA. A high degree for EE and DP could be shown for 18.5% (*n* = 204) of the study participants. Figure 1 shows, furthermore, that the percentage of participants with a high degree of EE and DP is greater for those who reported injuries or errors and adverse events. This is especially the case for the dimension DP where a high degree could be shown for 50% (reported injuries) and 44% (reported errors and adverse events) of the participants.

Comparisons were conducted for the domains injury and errors and adverse events. As nearly all respondents (99.4%) reported at least one factor of the domain safety comprising behavior a comparison between reported and not reported safety comprising behavior was impossible. Table 2 shows the frequency and the percentage (in brackets) of the negative safety outcomes as well as the greatest contributing factors for the domains error and adverse events and safety compromising behavior. Approximately one third of the participants (36.2%) reported that they experienced an injury in the last three months, 73.7% positively responded to one of the items measuring error or adverse events. Greatest contributing factors in the measure safety comprising behavior included speed limits and the check of the ambulance. For the measure error and adverse events factors are more diverse ranking from not printing and properly interpreting an EKG strip to not checking the glucose level in a patient with altered mental status.

**Table 2:** Number of negative safety outcomes measured by the EMS-SI

Safety outcome measures	N(%) N = 1101
Injury	
Yes	398 (36,2)
No	703 (63,9)
Error and adverse events	
Yes	811 (73,7)
No	290 (26,3)
Greatest contributing factors	
Did not print and properly interpret a 6 in. EKG strip	357 (44,0)
Made patient with chest pain ambulate instead of using stretcher	322 (39,7)
Did not administer necessary treatment for specific condition/malady	257 (31,7)
Did not establish an IV after two attempts	190 (23,4)
Did not check glucose level in a patient with altered mental status	135 (16,6)
Safety Comprising Behavior	
Yes	1094 (99,4)
No	7 (0,6)
Greatest contributing factors	
Greatly exceeded speed limit while responding lights and sirens	876 (80,0)
Exceeded speed limit while routinely driving	790 (72,2)
Did not complete pre-shift check of equipment and medications	760 (69,5)
Did not restock the ambulance before a call or shift	742 (67,8)
Overly stressed during a shift	728 (66,5)

Table 3 shows the Pearson correlation coefficients for the dimensions of the MBI and the domains of EMS-SI. All correlation coefficients are significant except the one between personal accomplishment and injury. Following Cohen [33] regarding the effect size of the Pearson correlation coefficient, a moderate and positive correlation can be shown between the measure safety comprising behavior and the dimensions EE and DP of the MBI.

**Table 3:** Pearson correlation coefficients for the MBI and the EMS-SI

	MBI	Emotional Exhaustion	Depersonalisation	Personal Accomplishment
EMS-SI				
Injury		0.2109*	0.1895*	− 0.0409
Error and Adverse Events		0.1605*	0.2679*	− 0.1562*
Safety Comprimising Behavior		0.4355*	0.4052*	− 0.1717*

* *p* < 0.01

### Regression results

The results of the regression models for the three safety outcomes are summarized in Table 4. For the safety outcomes injury and error and adverse events a logistic regression was conducted, consequently the odds ratios are presented. For the safety outcome safety compromising behavior a linear regression was used as a differentiation between those who reported safety compromising behavior and those who did not was not possible. Therefore, the regression coefficients are displayed in Table 4. The association between burnout and safety outcomes is assessed by the dimensions emotional exhaustion and depersonalization. We observed a positive and significant association between both dimensions and the three safety outcomes besides between emotional exhaustion and error and adverse events. Age is negatively associated with the safety outcomes (except the age category 40–49 for the safety outcomes injury). A positive association between experience in EMS and the safety outcomes injury and safety compromising behavior is observed. Satisfaction with the current job is negatively associated with the safety outcome safety compromising behavior, satisfaction with the professional status with the safety outcome safety compromising behavior and error and adverse events. We observed a negative association between no intention to leave the current job within the next year and all safety outcomes. Additionally, a negative effect between the safety compromising behavior and the recommendation of the EMS area as a good place to work is observed.

**Table 4:** Results of the regressions analysis

	Injury		Error and Adverse Events		Safety-Compromising Behavior	
	Odds Ratio	95% CI	Odds Ratio	95% CI	Coefficient	95% CI
Burnout						
Emotional Exhaustion	1.48225**	1.07–2.05	1.04878	.72–1.54	.892386***	.40–1.39
Depersonalization	1.53438***	1.16–2.03	1.57450***	1.13–2.19	1.20405***	.78–1.62
Age						
30–39	.721825*	.49–1.06	.570877***	.38–.87	−.908149***	−1.43 - -.38
40–49	.746867	.40–1.39	.371898***	.19–.71	−1.71182***	−2.59 - -.84
> =50	.342833**	.13–0.92	.296491**	.11–.80	−3.57810***	−4.91 - -2.25
Gender	1.05635	.72–1.54	.827610	.54–1.26	−.3577269	−.89–.17
Experience in EMS	1.04295**	1.01–1.08	1.00161	.97–1.03	.0453481**	.001–.09
Full-time	1.33335	.86–2.07	1.39728	.89–2.20	.2312638	−.41–.88
Satisfaction						
Satisfaction current job	1.15952	.85–1.59	1.24126	.88–1.76	−.4735402**	−.93 - -.01
Satisfaction professional status	.826897	.62–1.10	.666836***	.49–.91	−.833566***	−1.21 - -.45
Satisfaction Wages	1.01309	.73–1.40	1.22432	.86–1.74	−.2322153	−.66–.19
Intention to leave/Recommendation						
No intention to leave	.659769**	.47–0.92	.549649***	.38–.79	−.777390***	−1.24 - -.31
Recommendation	1.07947	.78–1.50	1.40473*	.97–2.02	−.656998***	−1.11 - -.20
Level of EMS-qualification						
Paramedic ‘Notfallsanitaeter’	.562515	.27–1.18	1.12939	.51–2.51	−.1906393	−1.35 - .97
Paramedic ‘Rettungsassistent’	.653700	.35–1.23	1.24601	.62–2.50	−.100563	−1.10 - .90
EMT I ‘Rettungssanitaeter’	.738753	.36–1.51	1.15101	.52–2.55	−.6527509	−1.77 - .46
Constant	.399562**	.16–.98	3.24927**	1.22–8.63	9.20007***	7.77–10.63

*** p < 0.01, ** *p* < 0.05, * *p* < 0.1

## Discussion

Burnout among health care workers is seen as an important issue in patient care. Several studies have analyzed the risk factors and potential consequences of the burnout syndrome for EMS-workers. Especially in the context of health threatening diseases medical errors and failure may lead to fatal consequences [34]. Burnout of health care professionals may pose a risk to patients’ safety and health outcomes. Therefore, this study focuses on investigating the association between burnout and safety outcomes by combining tools measuring the degree of burnout and adverse events (safety outcomes) for EMS-workers.

We got the result that between 19.9 and 40% of the participants showed a high degree of burnout in one of the burnout dimensions with the highest percentages of participants in the dimension depersonalization. Compared with the prevalence of burnout for nurses and surgeons presented in the introduction our results for depersonalization were in the higher range. A potential reason might be that with 86% the majority of our participants were male and men are more affected by depersonalization than women [35]. Our results are furthermore not fully in line with the results of the presented burnout studies for EMS-workers. The only German study [14] examining the degree of burnout using the MBI, got the result that the dimension with the highest percentage of participants with a high degree of burnout was personal accomplishment. However, the number of 98 considered questionnaires was considerably low and the analysis was limited to two regions in Germany. Alexander and Klein [18] could show for ambulance personnel in the UK that personal accomplishment is the dimension with the highest percentage of a high degree of burnout. In contrast Essex and Scott [15] showed the highest percentage of a high degree of burnout for the dimension depersonalization. They reported significantly higher scores than other studies (92% high level for EE, 99.3% high level for DP, and 76.1% high level for PA). A potential reason might be the combination of full or part-time job and EMS volunteering which may result in a double burden.

Regarding safety outcomes, the comparison of our results of the EMS-SI with the results by Weaver et al. [27] shows that a greater percentage of participants reported negative safety outcomes in all domains. A potential reason for difference might be various regional settings. Furthermore, the regression results show that burnout is significantly associated with the safety outcomes injury, error and adverse events, and safety-compromising behavior. This is especially in line with studies investigating the association of self-reported medical errors and self-reported burnout of healthcare staff [36–39]. One of those studies could furthermore show a mediating role of burnout for patient outcomes and job turnover indicating that the working environment supports psychological well-being and ensures patients safety [38].

Our survey shows that 46% of the EMS-workers are dissatisfied with their current job and 54% intend to leave their job within the next year. Compared to a study of Linda Aiken and colleagues [40], who investigated these items for nursing professionals, our results exceed the findings for nurses surveyed in Germany, 11 other European countries and the US (except for the item job satisfaction in Greece). Furthermore, they could show a significant association between satisfaction and intention to leave and the quality of care. Results of Patterson and colleagues who investigated job satisfaction of EMT-Basics and EMT-Paramedics showed that satisfaction varies across several important personal and organizational factors. They recommend a longitudinal study design to explore fully the importance of these factors in predicting job satisfaction [41].

In summary, job satisfaction, the intention to leave the current position and burnout in the EMS-workers workforce are critical issues as they pose safety risks for patients as well as EMS-workers. Given the increasing numbers of cases in prehospital emergency care, EMS managers should be aware of factors that affect the items such as the intention to leave a job. Therefore, identifying predictors of job satisfaction, intention to leave and burnout is an essential issue to ensure prehospital emergency care and improve its safety outcomes.

### Limitations

Several limitations need to be mentioned. Online surveys might lead to a selection bias, as people without internet access are not able to participate. Studies have shown that variables such as “high age group” and “female” are associated with less internet use [42]. Another limitation is that the calculation of a response rate is not possible. The study population is all EMS-workers in Germany. Since no standardized data on the number of EMS-workers on federal state level and information on the number of voluntary EMS-workers for Germany is available, a clear numerical number for the study population could not defined. However, the characteristics of our study population regarding gender and EMS-qualification is similar to nationwide data (77% are male and 71% have the EMS-qualification paramedic) [43, 44]. A further methodological limitation of the study findings might be the use of self-report measures for burnout and safety outcomes. Study participants with high burnout levels may over-report adverse events due to their negative emotional state [45]. Fahrenkopf and colleagues [46] used objective and subjective measures of error. They could show that burned out residents and non-burned out residents showed similar rates of error when using objective measurement. However, burned out residents reported a higher mean number of errors. Without taking into account the degree of burnout, prior studies have shown non-reporting rates of 4–19% for adverse events [47–49]. Reasons for underreporting might be a poor safety culture resulting in an unwillingness to report adverse events or that EMS-workers might not realize that an adverse event occurred. Finally, the cross-sectional study design prohibits determination of causality. Whether a high degree of burnout preceded negative safety oucomes cannot be analyzed.

## Conclusion

This first large German study analyzing burnout and safety outcomes could show that the share of EMS-workers with a high degree of burnout in one dimension varies between 20 and 40%. A high percentage of the surveyed EMS-workers reported at least one negative safety outcome in the three measures of the EMS-SI. It could be shown that burnout is significantly associated with safety outcomes. An expansion of the psychological support of EMS-workers in Germany seems to be necessary. Setting-based prevention could be a beneficial approach e.g. by enhancing the information flow between the EMS-workers and their management. Further analysis is needed to understand the nature and complex relations between various factors for example by using a structural equation model. Those further investigations have the potential to identify predictors for negative safety outcomes as well as determinants of burnout. Policymakers and managers of EMS should take care of the burnout prevalence and safety outcomes when qualifying EMS-workers and planning their working environments.

## Additional file

Additional file 1: Questionnaire. (DOCX 36 kb)

Additional file 2: EMS Qualification in Germany. (DOCX 17 kb)

## Abbreviations

DP: Depersonalization
EE: Emotional exhaustion
EKG: Electrocardiogram
EMS: Emergency medical services
EMS-SAQ: Emergency medical services-Safety Attitudes Questionnaire
EMS-SI: Emergency medical services-Safety Inventory
EMT: Emergency medical technician
MBI: Maslach Burnout Inventory
MBI-HSS: Maslach Burnout Inventory-Human Services Survey
PA: Personal Accomplishment
RN4CAST: Nurse forecasting in Europe
SD: Standard deviation
