# Supplementary material for: Burnout and safety outcomes - a cross-sectional nationwide survey of EMS-workers in Germany
Source: BMC Emerg Med. 2018 Aug 20;18:24. doi: 10.1186/s12873-018-0177-2 (PMC6102842; doi:10.1186/s12873-018-0177-2)
Supplement: Supplementary file 1 — Questionnaire. (DOCX 36 kb) [file 12873_2018_177_MOESM1_ESM.docx]

**Additional file 1**

**Appendix A – Questionnaire**

The English version of items, supplements, and scales is based on the used references in the text

Appendix A.1 Translation and adaption of the EMS-SAQ

| # of item in original EMS-SAQ questionnaire | **EMS-SAQ items** | **German adapted EMS-SAQ** |
| --- | --- | --- |
| 1 | I like my job. | Ich mag meine Arbeit. |
| 2 | EMS personnel input is well-received in this EMS. | In meinem Rettungsdienst werden Verbesserungsvorschläge der Mitarbeiter übernommen. |
| 3 | I would feel safe being treated by this EMS agency as a patient. | Ich würde mich sicher fühlen, wenn ich als Patient in meinem Rettungsdienstbereich versorgt werden würde. |
| 4 | Medical errors are handled appropriately at this EMS agency. | Mit medizinischen Fehlern wird in meinem Rettungsdienstbereich angemessen umgegangen. |
| 5 | This EMS agency does a good job of training new personnel. | Mein Rettungsdienstbereich bildet neue Mitarbeiter gut aus. |
| 6 | Working at this EMS agency is like being part of a large family. | In diesem Rettungsdienstbereich zu arbeiten, ist wie ein Teil einer großen Familie zu sein. |
| 7 | The management of this EMS agency supports my daily efforts. | Die Verwaltung meines Rettungsdienstbereichs unterstützt meine tägliche Arbeit. |
| 8 | I receive appropriate feedback about my performance. | Ich erhalte eine angemessene Rückmeldung über meine Leistung. |
| 9 | In this EMS agency, it is difficult to discuss errors. | In meinem Rettungsdienstbereich ist es schwierig Fehler anzusprechen. |
| 11 | This EMS agency is a good place to work. | Mein Rettungsdienstbereich ist ein guter Ort zum Arbeiten. |
| 12 | Management does not knowingly compromise the safety of patients. | Die Verwaltung gefährdet nicht wissentlich die Sicherheit der Patienten. |
| 13 | The levels of staffing at this EMS agency are sufficient to handle the number of calls. | Die personelle Besetzung des Rettungsdienstbereichs reicht aus, um die Anzahl der Anrufe abzuarbeiten. |
| 14 | I am encouraged by my colleagues to report any patient safety concerns I may have. | Ich bin durch meine Kollegen ermutigt, alle Bedenken in Hinsicht auf die Patientensicherheit zu melden. |
| 15 | The culture at this EMS agency makes it easy to learn from the errors of others. | Die Struktur des Rettungsdienstbereichs lässt es zu, aus den Fehlern der anderen zu lernen. |
| 16 | This EMS agency deals constructively with problem personnel. | In meinem Rettungsdienstbereich geht man mit problematischen Mitarbeiter/innen konstruktiv um. |
| 17 | At this EMS agency, it is difficult to speak up if I perceive a problem with patient care. | In meinem Rettungsdienstbereich ist es schwer, über beobachtete Probleme bei der Patientenversorgung zu sprechen. |
| 18 | When my workload becomes excessive, my performance is impaired. | Wenn mein Arbeitsaufkommen zu hoch ist, lässt meine Leistung nach. |
| 19 | I am provided with adequate, timely information about events that might affect my work. | Mir werden adäquate, zeitnahe Informationen über Ereignisse bereitgestellt, welche meine Arbeit beeinflussen könnten. |
| 22 | I know the proper channels to direct questions regarding patient safety. | Ich kenne die richtige Ansprechperson, um direkte Fragen zur Patientensicherheit zu stellen. |
| 23 | I am proud to work at this EMS agency. | Ich bin stolz in meinem Rettungsdienstbereich zu arbeiten. |
| 24 | Disagreements at this EMS agency are resolved appropriately (i.e., not who is right, but what is best for the patient). | Meinungsverschiedenheiten in meinem Rettungsdienstbereich werden angemessen gelöst (“nicht wer hat Recht, sondern was ist das Beste für den Patienten”). |
| 25 | I am less effective at work when fatigued. | Wenn ich müde werde, bin ich weniger effektiv. |
| 26 | I am more likely to make errors in tense or hostile situations. | Ich mache in angespannten und undurchsichtig Situationen am ehesten Fehler. |
| 27 | I have the support I need from other personnel to care for patients. | Ich bekomme von anderen an der Patientenversorgung beteiligten Mitarbeitern die Unterstützung, die ich benötige. |
| 28 | It is easy for personnel at this EMS agency to ask questions when there is something they do not understand. | Es ist für Mitarbeiter im Rettungsdienstbereich leicht Fragen zu stellen, wenn etwas nicht verstanden wird. |
| 29 | Personnel here work together as a well-coordinated team. | Die Mitarbeiter arbeiten als ein gut eingespieltes Team zusammen. |
| 31 | Morale at this EMS agency is high. | Die Arbeitsmoral in meinem Rettungsdienstbereich wird groß geschrieben. |
| 32 | Trainees in my discipline are adequately supervised. | Auszubildende in meinem Beruf werden gut betreut. |
| 34 | Fatigue impairs my performance during emergency situations. | Ermüdung beeinträchtigt meine Leistungsfähigkeit in Notfallsituationen. |
| 44 | All the necessary information for treating patients is routinely available to me. | Alle notwendigen Informationen zur Behandlung der Patienten stehen mir regelmäßig zur Verfügung. |
| **Supplement**  1. Items are grouped by dimensions:   - Safety Climate (7 items; questions 3, 4, 8, 9, 14, 15, 22), - Teamwork Climate (6 items; questions 2, 17, 24, 27 – 29), - Stress Recognition (4 items; questions 18, 25, 26, 34), - Perceptions of Management (4 items; questions 7, 12, 13, 19), - Working Conditions (4 items; questions 5, 16, 32, 44), - Job Satisfaction (5 items; questions 1, 6, 11, 23, 31).   2. Questions 9 and 17 are reverse coded to match the positive valence of the other questions.  3. The scale responses are assumed from the origin EMS-SAQ and coded to a 100-point scale:   - Absolutely disagree = 0, - Disagree very much = 20, - Somewhat disagree = 40, - Somewhat agree = 60, - Agree very much = 80, - Absolutely agree = 100.   4. Scores for each item are totaled and divided by the total number of items.  5. For each respondent a score ≥ 60 identified a positive and a score ≤ 60 identified a non-positive response. | | |

Appendix A.2 Translation and adaption of the EMS-SI

| # of item in original EMS-SI questionnaire | **EMS-SI items** | **German adapted EMS-SI** | **Scale** |
| --- | --- | --- | --- |
| 1 | I was injured during a shift. | Ich wurde während eines Dienstes verletzt. | A |
| 2 | I was overly stressed during a shift. | Ich war während einer Schicht außergewöhnlich gestresst. | A |
| 3 | I found myself at an unsafe scene. | Ich befand mich während eines Einsatzes an einem für mich unsicheren Ort. | A |
| 4 | I received a needle stick injury. | Ich hatte eine Nadelstichverletzung. | A |
| 5 | I may have been contaminated with copious amounts of patient bodily fluids. | Ich wurde mit einer größeren Menge Körperflüssigkeit eines Patienten kontaminiert. | A |
| 6 | I was involved in a collision involving one of my agency’s vehicles. | Ich war bei einer Kollision mit einem Fahrzeug des Rettungsdienstes 3beteiligt. | A |
| 7 | I have reported for my shift without getting adequate rest beforehand. | Ich habe meine Arbeit begonnen, ohne ausreichend Ruhepause gehabt zu haben. | A |
| 8 | I have reported for my shift after drinking alcohol within the previous 8 hours. | Ich habe meine Arbeite begonnen, obwohl ich innerhalb der letzten 8 Stunden Alkohol getrunken habe. | A |
| 9 | I did not complete a pre-shift check of equipment and medications because*…* | Ich habe den täglichen Check des Fahrzeuges, der Geräte und Medikamente nicht abgeschlossen, weil… | B |
| 10 | I did not restock the ambulance before a call or shift because… | Ich habe den Rettungswagen vor einem neuen Einsatz oder dem Dienstbeginn nicht aufgefüllt, weil… | B |
| 11 | I did not establish an IV after two attempts because*…* | Ich habe nach zwei Versuchen keinen IV-Zugang bekommen, weil… | B |
| 12 | I did not use a secondary treatment device when the preferred failed (e.g., IO instead of IV access, King airway instead of ET tube) because*…* | Ich habe eine zweite Behandlungsmöglichkeit nicht genutzt, nachdem die bevorzugte fehlgeschlagen war (z.B. IO-Zugang anstatt dem IV-Zugang, Larynxtubus anstatt Endotracheltubus), weil… | B |
| 13 | I did not check a glucose level in a patient with altered mental status because*…* | Ich habe den Zuckerwert bei einem Patienten mit Bewusstseinsstörungen nicht geprüft, weil… | B |
| 14 | I did not check a glucose level in a diabetic patient with nausea and vomiting because*…* | Ich habe den Zuckerwert bei einem bekannten Diabetes-Patienten mit Übelkeit und Erbrechen nicht geprüft, weil… | B |
| 15 | I did not perform an airway intervention (e.g., BVM, intubation, King/Combitube) on a patient with congestive heart failure while on route to the hospital because*…* | Ich habe eine Atemwegsintervention (z.B. Maske-Beutel-Beatmung, Intubation, Larynxtubus etc.) bei einem Patienten mit kongestiver Herzinsuffizienz während der Fahrt ins Krankenhaus nicht durchgeführt, weil… | B |
| 16 | I did not intubate a patient in respiratory arrest because*…* | Ich habe einen Patienten bei Atemstillstand nicht intubiert, weil… | B |
| 17 | I did not place a patient on the monitor because*…* | Ich habe den Patienten nicht an einen Monitor angeschlossen (Überwachung, RR, Puls, EKG), obwohl dieser es benötigte (vitale Bedrohung, v.A. Apoplex, Herzinfarkt etc.), weil… | B |
| 18 | I did not perform a 12-lead EKG on a patient with chest pain because*…* | Ich habe bei einem Patienten mit Brustschmerzen kein 12-Kanal-EKG durchgeführt, weil… | B |
| 19 | I did not perform a 12-lead EKG on a patient with STEMI because*…* | Ich habe bei einem Patienten mit einem ST-Hebungsinfarkt kein 12-Kanal-EKG durchgeführt, weil… | B |
| 20 | I confirmed a STEMI but did not administer aspirin when warranted because*…* | Ich habe einen ST-Hebungsinfarkt erkannt, aber kein Aspirin gegeben, obwohl es berechtigt war, weil… | B |
| 21 | I administered the wrong medication by not checking the label because*…* | Ich habe das falsche Medikament gegeben, da ich das Etikett nicht geprüft hatte, weil… | B |
| 22 | I administered the wrong dose of medication by not confirming the dose because*…* | Ich habe die falsche Dosis eines Medikamentes verabreicht, da ich mir die Dosis nicht habe bestätigen gelassen (z.B. Medikamentendosis durch die Verpackung, einen Kollegen oder Arzt bestätigt), weil… | B |
| 23 | I transferred a patient at the emergency department (ED) with an unrecognized esophageal intubation (ET tube placed in esophagus rather than trachea) because*…* | Ich habe in der Notaufnahme einen nicht erkannten Ösophagus intubierten Patienten (Endotrachealtubus lag in der Speiseröhre anstatt der Luftröhre) übergeben, weil… | B |
| 24 | I did not secure an embedded object in a wound instead of securing the object with bandage and accidently removed it because… | Ich habe ein Fremdkörper in der Wunde nicht mit einer Binde fixiert und diesen versehentlich entnommen, weil… | B |
| 25 | I did not print and properly interpret a 6 inch EKG strip because*…* | Ich habe einen EKG Streifen nicht ausgedruckt und interpretiert, obwohl ich das EKG angeschlossen hatte, weil… | B |
| 26 | I did not properly size a piece of equipment and then used it on a patient (e.g., ET tube, C-collar, airway adjunct, IV catheter) because*…* | Ich habe bei einem Patienten nicht die richtige Größe eines medizinischen Gerätes verwendet (z.B. Endotracheltubus, Stifneck, Atemwegshilfsmittel, IV Katheter), weil… | B |
| 27 | I did not transport a specialty care patient to a specialty care facility (i.e., trauma, stroke, pediatric) because… | Ich habe einen medizinisch-besonderen Patienten nicht an eine spezielle Behandlungseinrichtung (z.B. Trauma-Patient in Schockraum, Schlaganfall in Stroke Unit, Kinder in pädiatrische Klinik) transportiert, weil… | B |
| 28 | I accidentally started an IO in a location outside of protocol. | Ich habe versehentlich einen intraossären Zugang außerhalb des Algorithmus/Standard durchgeführt. | A |
| 29 | I made a patient with chest pain ambulate instead of using a stretcher. | Ich habe einen Patienten mit Brutschmerzen laufen lassen, anstelle die Fahrtrage oder einen Stuhl zu verwenden. | A |
| 30 | I did not administer the necessary treatment for a specific condition/malady. | Ich konnte eine notwendige Behandlung für einen besonderen Krankheitszustand nicht anwenden. | A |
| 31 | I placed an IV into an artery instead of into a vein. | Ich habe einen Zugang arteriell anstatt intravenös gelegt. | A |
| 32 | I accessed a dialysis port or other vascular device outside of protocol. | Ich habe außerhalb des Algorithmus/Standards auf einen Dialyse-Port oder einen anderen Gefäßzugang zugegriffen. | A |
| 33 | I accidentally dislodged an ET tube. | Ich habe versehentlich einen Endotracheltubus entfernt. | A |
| 34 | I accidentally dropped a patient while on a transportation device (i.e., stretcher, stair chair). | Ich habe beim Transport versehentlich einen Patienten fallen gelassen (z.B. Trage, Stuhl). | A |
| 35 | I accidentally caused physical injury to a patient moving the patient. | Ich habe bei der Bewegung des Patienten diesem versehentlich Verletzungen zugefügt. | A |
| 36 | I have “fudged” information on a patient care report (i.e., vitals, chronology of events). | Ich habe Informationen in einem Einsatzprotokoll frisiert (z.B. Vitalparameter, Ablauf der Ereignisse). | A |
| 37 | I felt vulnerable to harm due to lack of appropriate PPE (i.e., BSI, turnout gear, etc.). | Ich finde, dass ich wegen ungeeigneter persönlicher Schutzausrüstung ungeschützt bin (z.B. Handschutz, Winterkleidung, etc.). | A |
| 38 | I felt that **a patient’s** safety was jeopardized because my agency did not provide me with updated equipment. | Ich finde, dass die **Sicherheit des Patienten** durch fehlende Bereitstellung der aktuellsten/neuesten Ausrüstung gefährdet ist. | A |
| 39 | I felt that **my** safety was jeopardized because my agency did not provide me with updated equipment. | Ich finde, dass **meine Sicherheit** durch fehlende Bereitstellung der aktuellsten/neuesten Ausrüstung gefährdet ist. | A |
| 40 | I felt that **a patient’s** safety was jeopardized because my agency did not provide me with updated protocols/policies/procedures. | Ich finde, dass die **Sicherheit der Patienten** gefährdet ist, da mein Rettungsdienstbereich mir keine aktualisierten Standards/Algorithmen/Verfahren- bzw. Handlungsanweisungen vorgibt. | A |
| 41 | I felt that **my** safety was jeopardized because my agency did not provide me with updated protocols/policies/procedures. | Ich finde, dass **meine Sicherheit** gefährdet ist, da mein Rettungsdienstbereich mir keine aktualisierten Standards/Algorithmen/Verfahren- bzw. Handlungsanweisungen vorgibt. | A |
| 42 | I have exceeded the speed limit while routinely driving the unit in a non-emergency mode. | Ich habe die Geschwindigkeitsbegrenzung während einer Fahrt ohne Sonderrechte überschritten. | A |
| 43 | I have greatly exceeded the speed limit while responding lights and sirens (i.e., more than 15 mph over the posted speed limit). | Ich habe während der Fahrten mit Sonderrechten die Geschwindigkeitsbegrenzung überschritten (d.h. um mehr als 25 km/h über der angegebenen Begrenzung). | A |
| 44 | My “chute time” (time from call received to rolling) was greater than 1 minute. | Meine Ausrückzeit (Zeit von Alarm bis zum Beginn der Einsatzfahrt) war größer als 1 Minute. | A |
| **Supplement**  Items are grouped by dimensions:   - Injury (2 items; questions 1, 4), - Error or Adverse Event (25 items; questions 11 – 35), - Safety-Compromising Behavior (17 items; questions 2, 3, 5 – 10, 36 – 44). | | | |

Appendix A.3 Translation and adaption of the MBI

| # of item in original MBI questionnaire | **MBI items** | **German adapted MBI** |
| --- | --- | --- |
| 1 | I feel emotionally drained from my work. | Durch meine Arbeit fühle ich mich gefühlsmäßig am Ende. |
| 2 | I feel used up at the end of the workday. | Am Ende des Arbeitstages fühle ich mich erledigt. |
| 3 | I feel fatigued when I get up in the morning and have to face another day on the job. | Ich fühle mich schon müde, wenn ich morgens aufstehe und wieder ein Arbeitstag vor mir liegt. |
| 4 | I can easily understand how my recipients feel about things. | Es gelingt mir gut, mich in meine Patienten hineinzuversetzen. |
| 5 | I feel I treat some recipients as if they were impersonal ‘objects’. | Ich glaube, ich behandle Patienten zum Teil ziemlich unpersönlich. |
| 6 | Working with people all day is really a strain for me. | Den ganzen Tag mit Leuten zu arbeiten, stellt eine Belastung für mich da. |
| 7 | I deal very effectively with the problems of my recipients. | Mit den Problemen meiner Patienten kann ich gut umgehen. |
| 8 | I feel burned out from my work. | Durch meine Arbeit fühle ich mich ausgelaugt. |
| 9 | I feel I’m positively influencing other people’s lives through my work. | Ich glaube, dass ich das Leben anderer Leute durch meine Arbeit positiv beeinflussen kann. |
| 10 | I’ve become more callous towards people since I took this job. | Seit ich diese Arbeit mache, bin ich gleichgültiger gegenüber Menschen geworden. |
| 11 | I worry that this job is hardening me emotionally. | Ich fürchte, dass diese Arbeit mich emotional verhärtet. |
| 12 | I feel very energetic. | Ich fühle mich voller Tatkraft. |
| 13 | I feel frustrated by my job. | Meine Arbeit frustriert mich. |
| 14 | I feel I’m working too hard on my job. | Ich glaube, ich arbeite zu hart. |
| 15 | I don’t really care what happens to some recipients. | Bei manchen Patienten interessiert es mich eigentlich nicht wirklich, was mit ihnen wird. |
| 16 | Working with people directly puts too much stress on me. | Mit Menschen direkt zusammen zu arbeiten belastet mich zu sehr. |
| 17 | I can easily create a relaxed atmosphere with my recipients. | Es fällt mir leicht, eine entspannte Atmosphäre mit meinen Patienten herzustellen. |
| 18 | I have accomplished many worthwhile things in this job. | Ich habe viele wertvolle Dinge in meiner derzeitigen Arbeit erreicht. |
| 19 | I feel exhilarated after working closely with my recipients. | Ich fühle mich angeregt, wenn ich intensiv mit meinen Patienten gearbeitet habe. |
| 20 | I feel like I’m at the end of my rope. | Ich fühle mich am Ende. |
| 21 | In my work, I deal with emotional problems very calmly. | Ich gehe bei meiner Arbeit mit emotionalen Problemen sehr ruhig und gelassen um. |
| 22 | I feel recipients blame me for some of their problems. | Ich habe das Gefühl, Patienten geben mir die Schuld für einige ihrer Probleme. |
| **Supplement**  Items are grouped by dimensions:   - Emotional exhaustion (9 items; questions 1 – 3, 6, 8, 13, 14, 16, 20), - Depersonalization (5 items; questions 5, 10, 11, 15, 22), - Personal accomplishment (8 items; questions 4, 7, 9, 12, 17 – 19, 21). | | |

Appendix A.4 Required items of the RN4Cast study

| **RN4Cast items** | **German adapted EMS-version** | **Scale and answer options in German EMS-version** |
| --- | --- | --- |
| How satisfied are you with your current job in this hospital? | Wie zufrieden sind Sie alles in allem betrachtet mit Ihrem jetzigen Arbeitsplatz? | 4-point Likert (very dissatisfied, a little dissatisfied, moderately satisfied, very satisfied) |
| How satisfied are you with the following aspects of your job?   - Work schedule flexibility - Opportunities for advancement - Independence at work - Professional status - Wages - Educational opportunities - Annual leave - Sick leave - Study leave | Wie zufrieden sind Sie mit folgenden Aspekten Ihres Arbeitsplatzes?   - Flexibilität des Dienstplans - Selbstständigkeit bei der Arbeit - Beruflicher Status - Gehalt - Fort- und Weiterbildungsmöglichkeiten - Urlaubstage - Krankheitsregelung - Fortbildungsurlaub | 6-point Likert (extremely dissatisfied, very dissatisfied, somewhat dissatisfied, somewhat satisfied, very satisfied, extremely satisfied) |
| If possible, would you leave your current hospital within next year as a result of job dissatisfaction? | Wenn Sie die Möglichkeit hätten, würden Sie innerhalb des nächsten Jahres Ihren jetzigen Rettungsdienstbereich aufgrund von Unzufriedenheit am Arbeitsplatz verlassen? | Yes-no question (yes, no) |
| If yes, what type of work would you seek? | Falls ja, nach welcher Art von Arbeit würden Sie suchen? | Question with predetermined response options (EMS-workers in another EMS area, Medical activities in a company outside the EMS or in a hospital, An activity outside the EMS profession) |
| If you were looking for another job, how easy do you think it would be for you to find an acceptable job in nursing? | Wenn Sie eine neue Arbeitsstelle suchen würden: Wie einfach oder schwierig wäre es für Sie, eine zufriedenstellende Position/Stelle im Rettungsdienst zu finden? | 4-point Likert (very difficult, fairly difficult, fairly easy, very easy) |
| Would you recommend your hospital to a nurse colleague as a good place to work? | Würden Sie Ihren Rettungsdienstbereich als eine gute Arbeitsstelle weiterempfehlen? | 4-point Likert (no, probably no, probably yes, yes) |
| In general, how would you describe the quality of nursing care delivered to patients on your unit/ward? | Wie würden Sie insgesamt die Qualität der rettungsdienstlichen Maßnahmen den Patienten betreffend einstufen? | 4-point Likert (poor, fair, good, excellent, do not wish to answer) |
| The following questions ask for your opinion about patient safety issues in your employment setting.   - Staff feel like their mistakes are held against them. - Important patient care information is often lost during shift changes. - Things “fall between the cracks” when transferring patients from one unit to another. | In den folgenden Fragen werden Sie um Ihre Meinung zu Aspekten der Patientensicherheit an Ihrem Arbeitsplatz gebeten.   - Das Personal hat den Eindruck, dass Ihnen Fehler vorgehalten werden. - Informationen gehen bei der Schichtübergabe verloren. - Dinge „gehen unter“, wenn Patienten in der Notaufnahme übergeben werden. | 4-point Likert (strongly disagree, disagree, agree, strongly agree, do not wish to answer) |
| How often would you say each of the following incidents occurs involving you or your patients?   - Complaints from patients or their families - Verbal abuse toward nurses (by patients and/or families; by staff) - Physical abuse toward nurses (by patients and/or families; by staff) - Work related physical injuries to nurses | In den folgenden Fragen werden Sie um Ihre Meinung zu Aspekten der Patientensicherheit an Ihrem Arbeitsplatz gebeten.   - Beschwerden von Patienten und/oder Angehörigen - Beschimpfungen der Rettungsdienstkräfte durch Patienten und/oder deren Familienangehörige - Beschimpfungen der Rettungsdienstkräfte durch Kollegen - Handgreiflichkeiten gegenüber Rettungsdienstkräften durch Patienten und/oder deren Familienangehörige - Handgreiflichkeiten gegenüber Rettungsdienstkräften durch Kollegen - Arbeitsbedingte Verletzungen des Rettungsdienstpersonals | 7-point Likert (never, a few times a year or less, once a month or less, a few times a month, once a week, a few times a week, every day, do not wish to answer) |

Appendix A.5 Items and scale levels of individual characteristics

| **Item** | **Scale** | **Characteristics** |
| --- | --- | --- |
| Gender | Multiple choice (one answer) | Male, female |
| Age | Multiple choice (one answer) | ≤ 19, 20 – 24, 25 – 29, 30 – 34, 35-39, 40 – 44, 45 – 49, 50 – 54, ≥ 55 |
| Highest level of EMS apprenticeship | Multiple choice (one answer),  open question | Paramedic ‘Notfallsanitäter’, Paramedic ‘Rettungsassistent’, EMT-I ‘Rettungssanitäter’, EMT-B ‘Rettungshelfer’, medic, *open for input* |
| Age at highest level of EMS apprenticeship | Open question | *Open for input* [in years] |
| Work experience in EMS | Open question | *Open for input* [in years] |
| Work experience in current EMS area | Open question | *Open for input* [in years] |
| Satisfaction with occupational choice | 6-point Likert | Extremely dissatisfied, very dissatisfied, somewhat dissatisfied, somewhat satisfied, very satisfied, extremely satisfied |
| Employment relationship | Multiple choice (one answer) | Full-time, part-time (temporary incl.), voluntary work |
| Operating Range | Multiple choice (multiple answers) | Ambulance (emergency ambulance incl.), emergency doctor’s car, emergency rescue helicopter (intensive care helicopter incl.), call center, administration (e.g. quality assurance representative, rescue service commander) |
| Federal states | Dropdown | Baden-Württemberg, Bayern, Berlin, Brandenburg, Bremen, Hamburg, Hessen, Mecklenburg-Vorpommern, Niedersachsen, Nordrhein-Westfalen, Rheinland-Pfalz, Saarland, Sachsen, Sachsen-Anhalt, Schleswig-Holstein, Thüringen |
