# Supplementary material for: Burnout and safety outcomes - a cross-sectional nationwide survey of EMS-workers in Germany
Source: BMC Emerg Med. 2018 Aug 20;18:24. doi: 10.1186/s12873-018-0177-2 (PMC6102842; doi:10.1186/s12873-018-0177-2)
Supplement: Supplementary file 2 — EMS Qualification in Germany. (DOCX 17 kb) [file 12873_2018_177_MOESM2_ESM.docx]

**Additional file 2**

**Appendix B –EMS Qualification in Germany**

B.1 Level of EMS Qualification and content of apprenticeships in Germany

| **Level of EMS qualification in Germany** | **Content of apprenticeship** |
| --- | --- |
| ‘Rettungsassistent’ or ‘Notfallsanitäter’ | The apprenticeship of the ‘Rettungsassistent’ contains 1.200 hours of a theoretical and hands-on training course in a one year full-time program as well as a practical training in an ambulance station for another year full-time (1.600 hours).^A, B^  Since 01.01.2014 the occupational profile ‘Notfallsanitäter’ has been introduced. The apprenticeship has a length of three years full-time and amends the apprenticeship of the ‘Rettungsassistents’ by additional course content and practical training.^C^ |
| ‘Rettungssanitäter’ | A ‘Rettungssanitäter’ completes an apprenticeship consisting of 160 hours of theoretical and 160 hours of clinical education as well as 160 hours of practice in an ambulance station. It ends with a training course and an examination enduring 40 hours.^D,^ ^A^ |
| ‘Rettungshelfer’ | The ‘Rettungshelfer’ training includes 160 hours of theoretical and practical training and ends with an examination. In addition an internship for practice of 80 hours in an ambulance station need to be done and be completed in one year. An internship of 80 hours in a hospital is also recommended.^A^ |
| References: ^A^ Deutsches Rotes Kreuz, Generalsektretariat, Team 23, “Ordnung für Aus-, Fort- und Weiterbildung des Deutschen Roten Kreuzes, Teil: Rettungsdienst (Notfallrettung und Krankentransport) 2006,” 2005. ^B^ Bundesministerium der Justiz und Verbraucherschutz, Gesetz über den Beruf der Rettungsassistentin und des Rettungsassistenten (Rettungsassistentengesetz - RettAssG). ^C^ Bundesministerium der Justiz und Verbraucherschutz, Gesetz über den Beruf der Notfallsanitäterin und des Notfallsanitäters (Notfallsanitätergesetz - NotSanG).  ^D^ Bund/Länderausschuß Rettungswesen, “Grundsätze zur Ausbildung des Personals im Rettungsdienst,” 1977. [Online]. Available: http://www.notfallrettung.com/recht/rettsan/. [Accessed: 11-Nov-2016]. ^E^ M. A. Sucher and J. L. Waxler, “EMS Providers and System Roles,” in Principles of EMS Systems, 3rd ed., Jones & Bartlett Publ Inc, 2005, pp. 302–304. | |
